# Editor's Letter-Box

**Published:** 1899-12-23

**Authors:** 


### EDITOR'S LETTER-BOX.

[Our Correspondents are reminded that prolixity is a great bar to publication, and that brevity of style and conciseness of statement greatly facilitate early insertion.]

Dr. Urquhart writes: In your review of the report o the Northampton County Asylum you say that "It is wel known that this is one of the asylums in which is given a three years' course of instruction in all departments o nursing." As one of those who have the misfortune not to know, may I ask you to kindly explain how this is done How does Dr. Harding provide for instruction in the nursing of surgical, fever, and obstetric cases? And, further, what guarantee have the public that the certificate so gained is "really valuable"? What methods are adopted by independent examination to insure that a standard of efficiency is reached? At a time when the General Medical Council have decided that "it is contrary to the interests of the public to have two competing examining boards sitting in London," and when so many eminent medical men declare themselves in favour of the "one portal" system, I would ask if you are of opinion that it is in the public interest to promulgate opposite ideas as to certificates of competency in nursing.

*** In the year 1897 this subject was so well discussed in our columns that it seems hardly necessary to re-open it; but we may answer Dr. Urquhart's questions as follows: (1) We understand the Northampton system to involve a three years' course of instruction; that in the first year being chiefly devoted to surgical nursing, in the second chiefly to medical nursing, and the third chiefly to mental nursing. Examinations are held at the end of each year. (2) The guarantee which the public receives will always consist in the reputation of the asylum, precisely as is the case in general hospitals. (3) The question of competing examining boards for medical qualifications has nothing whatever to do with mental or other nursing certificates at present, because there is no legally constituted board for granting them.—Ed. *T.H.*

